# The regulation of animal behavior by cellular stress responses

**DOI:** 10.1016/j.yexcr.2021.112720

**Published:** 2021-08-15

**Authors:** Neşem P. Özbey, Maximilian A. Thompson, Rebecca C. Taylor

**Affiliations:** Neurobiology Division, MRC Laboratory of Molecular Biology, Cambridge, CB2 0QH, United Kingdom

**Keywords:** Stress response, UPR, Signaling, Behavior, Transcription factor

## Abstract

Cellular stress responses exist to detect the effects of stress on cells, and to activate protective mechanisms that promote resilience. As well as acting at the cellular level, stress response pathways can also regulate whole organism responses to stress. One way in which animals facilitate their survival in stressful environments is through behavioral adaptation; this review considers the evidence that activation of cellular stress responses plays an important role in mediating the changes to behavior that promote organismal survival upon stress.

## 1 Introduction

To ensure their survival, organisms require the ability to respond to changes in environmental conditions. These changes are often perceived through cellular stress, the perturbed homeostasis and accumulated cellular damage that results from altered environmental parameters or internal conditions that impose changed demands upon cells. To survive this stress, cells have evolved means to detect damage and homeostatic imbalance, and to enact mechanisms that restore equilibrium as well as adapt the organism to the changed environment [1].

These detection/response mechanisms are referred to as cellular stress responses. They are pathways that act in different cellular compartments to respond both independently and interconnectedly to homeostatic imbalance induced by different sources of cellular stress. They are frequently able to detect and respond to the presence of misfolded proteins, the accumulation of which is commonly caused by stressors including temperature fluctuation and the presence of xenobiotics [2]. However, stress responses can also respond to other forms of homeostatic imbalance, such as lipid perturbation. In addition, stress response mechanisms can be active under basal conditions, and have constitutive roles in the cell even in the absence of external stress [3].

Organelle-specific stress responses include the cytosolic heat shock response (HSR), and the unfolded protein responses of the endoplasmic reticulum (UPR^ER^) and mitochondria (UPR^mito^) [2]. In addition, other stress responses are attuned to the presence of specific stressors, such as the hypoxic stress response and the oxidative stress response, which respond to low oxygen levels and the presence of oxidative stress, respectively. Each of these responses utilizes upstream detector molecules to respond to the effects of stress or the presence of a stress-inducing agent. This then activates downstream signal transduction pathways that lead to the activation of effector mechanisms designed to protect from and repair the effects of stress, while enabling adaptation of the organism to new environmental conditions [2].

Many of the outputs of these stress responses act to improve the protein homeostasis (proteostasis) environment of the cell, through increases in protein folding capacity, protein degradation, and reduced levels of protein translation [2]. In addition, stress responses also regulate other means of adapting to stress: for example, HSR activation induces cytoskeletal alterations that may aid cellular transport, and UPR^ER^ activation changes the expression of many genes involved in metabolism and neuronal signaling, while UPR^mito^ activation upregulates genes involved in the immune response [[3], [4], [5], [6], [7]]. The ultimate organismal effects of stress response activation include improved resistance to stress and enhanced lifespan, although these different outputs are context-dependent and can be uncoupled [8,9].

As well as acting at the level of the cell, stress responses can also promote whole organism responses to stress. They can be activated by inter-tissue signaling, which coordinates their activation among the cells of an organism and allows different tissues to respond to stress in ways that ensure the survival of the whole animal [10]. Having a range of tissue-specific stress response outputs enables the organism to not only deal with the proteostatic consequences of stress, but also to facilitate other forms of long-term organismal adaptation to environmental change.

**Fig. 1:**
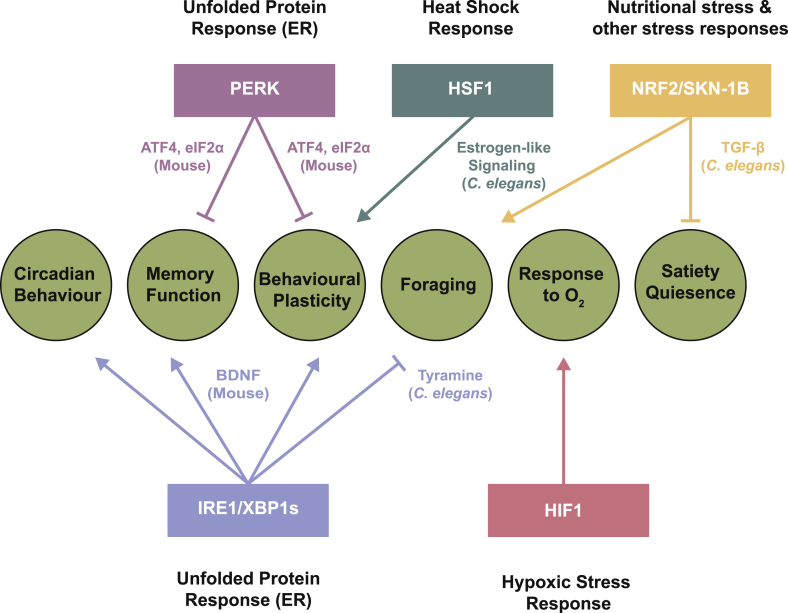
Regulation of behavior by cellular stress responses. Stress responses and their regulators (colored boxes) linked to the aspects of behavior they have been shown to regulate (green circles), annotated with molecular mediators where known.

As early as 1943, Curt Richter wrote about the importance of behavioral change to the maintenance of internal homeostasis [11]. Behavioral responses to a range of stresses have been documented, including changes to learning and locomotory behaviors. These enable organisms to avoid the source, and therefore lessen the effects of stressors. They can also promote altered feeding, reproductive or other strategies that allow long-term survival in the new environment. However, despite this evidence, the intersection between cellular and behavioral responses to stress and environmental change has been relatively unexplored. Given that cellular stress responses are designed to respond sensitively to the detection of stress, with a range of varied outputs enabling survival in stressful environments, it is interesting to consider whether they are also involved in mediating these behavioral responses to stress. Evidence suggests that, in at least some cases, they can be. This review will outline some ways in which cellular stress responses have been shown to coordinate whole organism behavioral changes that aim to facilitate survival in stressful environments (Fig. 1).

### 1.1 Heat shock response

The HSR is a cytosolic stress response that responds to a variety of stressors in addition to heat, including pathogen infection, heavy metals, and the expression of disease-associated misfolded proteins [12,13]. Activation of the HSR is dependent upon a conserved transcription factor, heat shock factor 1 (HSF1), which can be activated upon heat shock in a cell-autonomous manner. Stress-induced misfolded proteins titrate away inhibitory chaperones, such as HSP90, that bind to HSF1, allowing monomeric HSF1 to trimerize and bind DNA. HSF1 is further regulated by numerous post-translational modifications and by cooperation with transcriptional cofactors, allowing specific and tight regulation of the HSR. HSF1 target genes include not only many chaperones, which promote protein folding, but also genes involved in a range of other processes, including cytoskeletal maintenance, growth, metabolism, and autophagy [4,[12], [13], [14]].

While for a long time this intracellular mode of HSF1 activation was seen as sufficient for activation of the HSR, recent evidence suggests that the HSR can also be activated by signals between tissues, coordinated by the nervous system. A pivotal study demonstrated that a pair of neurons in *C. elegans*, the AFD neurons that regulate thermosensory behavior, are required for activation of the HSR in peripheral tissues in response to heat, demonstrating a role for the nervous system as a master regulator of stress response activation [15]. This regulatory role requires GCY-8, a guanylyl cyclase exclusively expressed in the AFD neurons, and these neurons also regulate the response to misfolded proteins present within other tissues [16]. Neuronal control of the HSR involves serotonin release, and a GPCR, GTR-1, expressed in thermoregulatory chemosensory neurons has also been implicated, suggesting the involvement of multiple neuronal subtypes [17,18]. In addition, serotonergic signaling upon stress can also induce HSF1-mediated stress resistance in progeny [19]. HSR activation can therefore be mediated in an inter-tissue manner, via serotonin, enabling organism-wide stress resistance. Does this organism-wide response facilitate stress-responsive behavioral change?

In *C. elegans,* HSR activation can indeed participate in the control of temperature-regulated behavior. Heat stress-induced activation of HSF1 is able to modify behavioral plasticity, through estrogen-mediated cell non-autonomous regulation of thermosensory neurons [20]. In *hsf-1* mutants defective in thermotactic migration – movement towards cultivation temperature – expression of HSF-1 in muscles or the intestine can rescue this defect, through regulation of downstream genes including nuclear hormone receptors that are involved in estrogen-like signaling. These signals to the thermotactic neuronal circuit promote appropriate thermotactic behavior [20].

In addition, it has been shown in mammals that the HSR can be activated by stress that also invokes behavioral alterations, demonstrating a correlation between the activation of this stress response and the promotion of behavioral change. Restraint stress, for example, which leads to the activation of a neurohormonal stress response mediated by the hypothalamic-pituitary-adrenal axis, and to behavioral changes that include alterations in locomotor activity, causes HSF1 activation in the adrenal tissue of rats [21]. Data in both vertebrates and invertebrates therefore suggest that the HSR may function to modulate behavioral responses to stress, through inter-tissue signaling.

### 1.2 Unfolded protein response (ER)

Another organelle-specific stress response is the UPR^ER^, which is activated by stresses that impinge upon homeostasis in the endoplasmic reticulum (ER). These include perturbations in ER-localized protein folding, ER lipid imbalance, ER calcium depletion, and pathogenesis [22]. The three pathways that comprise the UPR^ER^ each include an upstream sensor molecule located in the ER membrane – IRE1, PERK and ATF6 - that activates downstream effector molecules to trigger homeostatic mechanisms that include transcriptional regulation, translational regulation, and RNA degradation, mitigating the effects of stress [22,23]. The UPR^ER^ has an acute phase that alleviates the short-term effects of stress, but it can also activate responses to chronic stress that include long-term adaptation and cell fate decisions such as the initiation of cell death programmes [24]. In addition, the UPR^ER^ acts during development to establish tissue differentiation, and plays a constitutive role under unstressed conditions to maintain homeostasis within the secretory pathway [3].

The UPR^ER^ is activated cell-autonomously through its interactions with the ER-localized HSP70 chaperone BiP. BiP is bound under unstressed conditions to the membrane-localized sensor molecules of the UPR^ER^, inhibiting their activation. Titration of BiP away from these sensors through its binding to stress-induced misfolded proteins allows the UPR^ER^ to become activated [23]. However, recent evidence suggests that the UPR^ER^ can also be activated by inter-tissue signals, leading to changes in how the organism responds to stressful environments. A GPCR, OCTR-1, may function in the ASH and ASI sensory neurons of *C. elegans* to inhibit UPR^ER^ activation in other tissues: upon mutation of OCTR-1, both the canonical UPR^ER^ as well as genes proposed to act as a non-canonical mechanism of ER stress resistance are activated, and resistance to pathogenesis is improved [25,26]. In addition, nervous system-specific expression of the spliced and active form of the XBP-1 transcription factor, XBP-1s, which is spliced by and acts downstream of the endoribonuclease IRE-1 upon UPR^ER^ activation, is sufficient to induce UPR^ER^ activation in the intestine, through the release of a secreted signal from neuronal vesicles, or from glial cells [27,28]. Importantly, this communication of UPR^ER^ activation leads to increased lifespan and stress resistance. These systemic changes are mediated by the activation of intestinal lysosomes and enhancement of lipid metabolism when the distal UPR^ER^ is activated by neuronal signals, and these effects require synthesis of the biogenic amine tyramine [5,6,29,30], whereas the effects of UPR^ER^ activation by glial cells require neuropeptide release [27].

Neuronal function involves many aspects of secretory pathway activity, suggesting that the UPR^ER^ is likely to be important in maintaining a functional nervous system. Indeed, the UPR is particularly active in neurons, and is responsible for the expression of uniquely neuronal proteins; this stress response has also been shown to play a range of roles in brain function, including the regulation of memory, learning and behavior [6,31,32]. Active translation, regulated by eIF2α which is phosphorylated by the UPR^ER^ regulator PERK, is required for the storing of memories, and reduced activity of PERK can enhance memory function [[33], [34], [35]]. eIF2α phosphorylation has also been implicated in the regulation of synaptic plasticity, and reduced PERK expression has been associated with changes in behavioral flexibility [[34], [35], [36]]. In addition, ATF4, which acts downstream of eIF2α, plays roles in both memory and plasticity [33,34,37]. Therefore, activation of the PERK branch of the UPR^ER^ has the potential to affect both memory formation and plasticity and, in turn, exert changes to behavior.

XBP1 has also been implicated in the regulation of memory formation [38]. Mice deficient in XBP1 show memory defects, while hippocampal expression of the spliced and active form of XBP1, XBP1s, increases the amplitude of excitatory postsynaptic potentials in long-term potentiation, and enhances performance in a range of memory-related tasks. XBP1 has also been shown to regulate the expression of a range of synaptic plasticity and memory-related genes, including brain-derived neurotrophic factor (BDNF), overexpression of which can rescue the memory deficits of XBP1-deficient animals [38]. In turn, BDNF signaling can regulate XBP1 levels and splicing, facilitating neurite growth [39]. While it is possible that these effects result from non-UPR^ER^-associated XBP1 function, the findings highlight that activation of the IRE1-XBP1 UPR^ER^ axis has the potential to modulate learning and memory in mammals.

Psychological stressors have been associated with increased levels of UPR^ER^ markers in the nervous system – for example, behavioral stress induces XBP1 splicing in the brain of mice and rats [40,41]. In addition, psychological stress causes the release of corticosteroids, and high levels of plasma corticosterone caused by interventions such as restraint stress correlate with elevated levels of UPR^ER^-inducible chaperones such as BiP in the hippocampus and cortex of mice [42]. Treatment with the anti-depressant luteolin reduces levels of these chaperones, and in fact elevated levels of ER stress-related genes have also been detected in the temporal cortex of human subjects with major depressive disorder [43]. The activation of IRE1α seen in the brains of mice subjected to mild behavioral stress can differ in localization, with IRE1α activity in different brain regions correlated with differing behaviors; temporal lobe activation has been associated with anxious neophobic behavior, while ventral striatum activation coincides with greater curiosity [40]. Together, this correlative evidence may implicate the activation of the UPR^ER^ within the brain in the emergence of stress-responsive behaviors.

In fact, some evidence suggests that UPR^ER^ activation may play a causative role in behavioral change. A polymorphism in the XBP1 promoter affecting its expression has been associated with the development of a range of psychological disorders, suggesting that UPR^ER^ activation may play a driving role in the etiology of psychiatric disease in humans [44]. In rats, infusion of the ER stress-inducing drug tunicamycin into the hippocampus leads to depressive behavior, accompanied by increased expression of UPR-related genes in the hippocampus, suggesting that UPR activation in the brain may not only correlate with but might in fact drive behavioral change [45]. In addition, suppression of eIF2α phosphorylation by treatment with the integrated stress response inhibitor ISRIB reduces anxiety in a mouse model of neuropsychiatric disease susceptibility [46]. Therefore, specific activation or inhibition of different UPR^ER^ branches in the brain can drive stress-associated changes in behavior.

In *C. elegans*, activation of the UPR^ER^ in the nervous system has recently been shown to promote changes to feeding and foraging behavior [6]. Pan-neuronal expression of *xbp-1s* drives a broad transcriptional remodelling of neuronal signaling, and leads to decreases in the probability of leaving a food source and in the degree and range of foraging, dependent upon synthesis of the biogenic amine tyramine. As nutrient deprivation induces *xbp-1* splicing in *C. elegans* neurons, including the RIM and RIC interneurons that synthesize tyramine, it is possible that the presence of neuronal spliced *xbp-1s* acts as an indicator of low nutrient conditions, and that constitutive *xbp-1s* expression inhibits the accurate perception of an animal's nutritional environment. This leads to *xbp-1s*-induced starvation-appropriate changes in a variety of feeding behaviors, including a decreased propensity to leave a food source, which is partially dependent upon the tyramine receptor *ser-2* [6]. This demonstrates that UPR^ER^ activation can directly drive changes to behaviors that respond to stress and increase organismal fitness. Interestingly, this is echoed in the finding that overexpression of Xbp1s in the POMC neurons of the murine hypothalamus drives increased locomotor activity, as well as protecting against diet-induced obesity via metabolic changes involving the liver, suggesting that activation of this proteostasis mechanism in the hypothalamus coordinately affects both motor behavior and metabolism [47].

Finally, another aspect of behavioral regulation that involves activation of the UPR^ER^ is that imposed by the circadian clock. This controls the coordination of physiology according to daily cycles, regulating the sleep/wake cycle as well as other aspects of metabolism and behavior that oscillate with daily environmental changes. The circadian clock regulates UPR^ER^ activation, with downstream effects on metabolism [[48], [49], [50]]. In turn, UPR^ER^ regulators including PERK, ATF4 and Xbp1 can themselves regulate aspects of circadian oscillations [[51], [52], [53]]. This means that circadian behaviors can be influenced by UPR^ER^ activation, and indeed, driving expression of Xbp1s in POMC neurons of the murine hypothalamus leads to increased circadian movement, suggesting another level of behavioral regulation imposed by UPR^ER^ activation [47].

### 1.3 Hypoxic stress response

Hypoxia is an important threat to homeostasis, and survival in most organisms depends upon the ability to adapt to fluctuating oxygen levels. The response to hypoxic stress is coordinated by hypoxia-inducible transcription factors (HIFs), which are stabilised by declining oxygen levels [54]. HIFs generally act as heterodimers, with a regulated HIF-α and a constitutive HIF-β subunit. In conditions of normoxia, HIFs are subject to proteasomal degradation; however, when oxygen levels are reduced, degradation is inhibited. This regulation is mediated by oxygen-dependent prolylhydroxylase-domain enzymes (PHDs) that lead to HIF degradation via the activity of the von Hippel–Lindau tumor suppressor protein (VHL); upon hypoxia, PHD activity is diminished and HIFα is stabilised [54,55]. The stabilised HIF heterodimer then binds to the promoter of target genes via hypoxia response elements (HREs) with the consensus sequence G/ACGTG [54]. These target genes regulate a huge array of physiological processes that together enable adaptation to hypoxia, including metabolism, vascular biology, cell survival and proliferation; and this transcriptional response also plays roles in other protective processes, such as immunity [56].

As well as acting cell autonomously, HIF activity may also be able to coordinate responses to hypoxia cell non-autonomously. In *C. elegans*, stabilization of HIF-1 can lead to increased longevity, and this effect on lifespan can be mediated through HIF-1-generated signals sent between tissues; neuronal stabilization of HIF-1 influences ageing through the upregulation of a single HIF-1 target gene, the hypoxia-responsive flavin-containing monooxygenase FMO-2, in the intestine [57,58]. Over-expression of *fmo-2* in the intestine is sufficient to induce lifespan extension, and its inter-tissue activation by neuronal HIF-1 is mediated by a proposed signaling pathway involving serotonin and the SER-7 serotonin receptor.

Behavioral alteration is an important and conserved element of the response to altered oxygen levels, and the HIF-1 hypoxic stress response may play a role in this coordination of behavior. In *C. elegans,* avoidance of hyperoxia (>14% O_2_) is mediated by a distributed network of sensory neurons, and is modulated by the presence of food and by genetic background [59,60]. Long term exposure to hypoxia, sufficient to lead to HIF-1 activation, changes the behavioral response of wild-type worms, leading to a preference for lower oxygen levels and the avoidance of hyperoxia when food is available (rather than just in its absence) [61]. This modified behavior depends upon the PHD-mediated stabilization of HIF-1 in neurons as well as in nonneuronal secretory cells of the somatic gonad, the tyraminergic uterine vulval cells (uv1), suggesting that the behavioral response to environmental oxygen also involves inputs from a cell type that belongs to the reproductive system. Stabilization of HIF-1 leads to a dramatic reorganization and simplification of the neuronal circuit for oxygen preference, with only the URX neurons absolutely required for hyperoxia avoidance [61].

HIF1 may also play roles in the mammalian behavioral response to hypoxia. Heterozygous mutation of HIF1α in mice leads to defective carotid body function in low oxygen levels, and impaired ventilatory adaptation, or alterations in breathing movement, as well as deficiencies in physiological responses when subjected to chronic hypoxia [62]. This suggests that the hypoxic stress response participates in the modification of behavioral responses to oxygen levels in multiple organisms, integrating these with the physiological response to hypoxia.

### 1.4 NRF/SKN-1-regulated stress responses

The NRF (Nuclear Factor E2-Related Factor) family of transcription factors is involved in the regulation of multiple stress responses. The best studied of these transcription factors, Nrf2, plays a critical role in the response to oxidative stress and xenobiotic stress through its regulation of the phase II detoxification system [63]. In unstressed conditions, Nrf2 is principally sequestered and degraded in the cytoplasm by its association with Keap1, which acts to facilitate the ubiquitination of cytosolic Nrf2. Upon the onset of Nrf2-activating conditions this interaction is disrupted, and Nrf2 translocates into the nucleus where it associates with small Maf proteins and binds to the ARE (antioxidant response element) to induce its targets, resulting in the activation of an array of antioxidant and detoxificant genes [63,64]. Nrf1 is also known to have roles in the oxidative stress response, but in addition it responds to proteosome dysfunction by upregulating proteosome subunit genes in human cells when proteosomes are pharmacologically inhibited [[63], [64], [65], [66]].

SKN-1 is the sole *C. elegans* homologue of the NRF family. It appears that the major functions of mammalian NRFs are performed by isoforms of SKN-1, rather than by separate genes as in mammalian systems [67]. Thus, various SKN-1 isoforms respond to oxidative stress, disrupted proteostasis, starvation, and dietary restriction [[68], [69], [70], [71], [72]]. Isoforms also show tissue specific expression patterns, with SKN-1A and SKN-1C expressed primarily in the intestine, and SKN-1B expressed in the ASI neurons [69]. SKN-1A is a functional homologue of Nrf1 and responds to proteasome dysfunction and unfolded proteins by upregulating proteasome subunits [65,71]. The best studied isoform, SKN-1C, acts in a manner analogous to Nrf2 as a central mediator of the phase 2 detoxification response and is required for organismal survival in conditions of oxidative or xenobiotic stress [67].

As well as activation by these stressors, *skn-1a/c* can be regulated hormonally by inter-tissue insulin/IGF1 like signaling (IIS), functioning in parallel with *daf-16/*FOXO downstream of *daf-2/*IGF1R and required for the full longevity resulting from reduced IIS [73]. SKN-1B, in contrast to the A and C isoforms is expressed specifically in the chemosensory ASI neurons and lacks functionally important features of other SKN-1 isoforms including the crucial DIDLID motif [67]. This isoform has also been implicated in the remodelling of mitochondrial networks in another tissue, the muscle, suggesting that cell non-autonomous signaling downstream of SKN-1B has profound physiological effects in a distant tissue [74]. Thus, SKN-1 responds to a broad range of stresses in an isoform specific manner.

SKN-1 is important for the longevity resulting from dietary restriction, and SKN-1B is expressed in the chemosensory ASI neurons. This suggests the potential for SKN-1B to act in the behavioural response to changing food levels as well the physiological response to this challenge. Indeed, foraging behaviour is impaired in animals with null mutations in *skn-1*, with *skn-1* animals remaining on limited food when wild-type animals would instead choose to forage for more abundant food [75]. Recent work directly attributes this reduction in exploratory behaviour to SKN-1B, demonstrating that a null allele specific for that isoform recapitulates this foraging defect and that transgenic rescue of *skn-1b* rescues foraging behaviours [74].

When fasted *C. elegans* are re-fed, they enter a state termed satiety induced quiescence, in which they cease pharyngeal pumping and movement. Animals lacking *skn-1b* quiesce for longer than wildtype under these conditions, suggesting that this behaviour is also modulated by *skn-1* [74]. Ablation of the ASI neurons has the opposite effect on satiety quiescence, suggesting that this is not a consequence of cell death or inactivity of the ASI neurons [76]. Furthermore, *skn-1b* animals preferentially remain on higher quality food sources, suggesting that they retain the ability to sense food. This points towards a role for *skn-1b* in enacting a behavioural response to changes in chemosensory information about food levels, rather than simply an inability to sense food. Notably, the secreted neuropeptide DAF-7/TGF-B is known to promote quiescence and *daf-7* mutants do not exhibit satiety quiescence behaviour. SKN-1B requires DAF-7 in order to promote satiety induced quiescence, and expression of *daf-7* is increased in *skn-1b* null animals. SKN-1B also regulates the secretion of insulin like peptides in order to regulate the amount of food that animals consume, and interacts with the regulation of quiescence resulting from reduced insulin signaling [74]. Therefore, neuropeptide signaling downstream of SKN-1B regulates multiple aspects of the organismal behavioural response to food in parallel to major physiological changes including mitochondrial remodelling and enabling dietary restriction induced longevity. Whether other SKN-1-activating stresses also induce behavioural changes remains an open question in *C. elegans*.

Nrf2 may play a conserved role in satiety-related behaviour in mammals, as Nrf2 is present in multiple brain tissues including the human hypothalamus, which plays an important role in mammalian satiety responses, and in the olfactory bulb of the mouse brain at significantly enriched concentrations [75]. Nrfs are also known to influence other aspects of mammalian behaviour, with Nrf2 expression associated with various depression-like behaviours in different animal models. Higher Nrf2 levels confer a degree of resistance to learned helplessness in rats, and Nrf2 has been implicated in anxiety-like behaviours in rats as well as in seasonal depression-like behaviour in Medaka fish [[77], [78], [79]]. Nrf2-deficient mice also display altered behaviour as well as disrupted monoamine signaling [80]. SKN-1/Nrf mediated stress responses therefore influence behaviour in numerous systems of varying complexity, in parallel to mediating cellular and organismal physiological responses to stresses.

## 2 Conclusions

These findings suggest that stressful environmental conditions which lead to the disruption of cellular homeostasis can activate stress response mechanisms that both increase the cellular ability to withstand and adapt to this disruption of homeostasis, and promote behavioral strategies to avoid these conditions and lessen their impact on the organism (Fig. 1). Such behavioral effects represent an underappreciated output of cellular stress responses that are better known for their roles in the regulation of cellular proteostasis; the regulation of behavior helps them to facilitate long-term adaptation to environmental conditions. Our understanding of these behavioural roles of stress responses is clearly still in its infancy, with much of the current evidence correlative in nature, and there are many interesting avenues for further exploration. For example, do other stress responses, such as the UPR^mito^, also modulate organismal behavior? Are there other behaviors, beyond those discussed here, that can be influenced by cellular stress response activation? And are the behavioural effects of stress response activation always coupled with improvements in proteostasis and other forms of cellular homeostasis, or can these effector mechanisms be uncoupled? Better understanding of this aspect of stress response output will expand our appreciation of these pathways, and the organismal roles they play in surviving environmental stress.

## Credit author statement

N.P.O., M.A.T., and R.C.T. wrote and reviewed the manuscript.

## References

[1] Kotas M.E., Medzhitov R. (2015). Homeostasis, inflammation, and disease susceptibility. Cell.

[2] Sala A.J., Bott L.C., Morimoto R.I. (2017). Shaping proteostasis at the cellular, tissue, and organismal level. J. Cell Biol..

[3] Acosta-Alvear D., Zhou Y., Blais A., Tsikitis M., Lents N.H., Arias C., Lennon C.J., Kluger Y., Dynlacht B.D. (2007). XBP1 controls diverse cell type- and condition-specific transcriptional regulatory networks. Mol. Cell.

[4] Baird N.A., Douglas P.M., Simic M.S., Grant A.R., Moresco J.J., Wolff S.C., Yates J.R., Manning G., Dillin A. (2014). HSF-1-mediated cytoskeletal integrity determines thermotolerance and life span. Science.

[5] Imanikia S., Ozbey N.P., Krueger C., Casanueva M.O., Taylor R.C. (2019). Neuronal XBP-1 activates intestinal lysosomes to improve proteostasis in C. elegans. Curr. Biol..

[6] Ozbey N.P., Imanikia S., Krueger C., Hardege I., Morud J., Sheng M., Schafer W.R., Casanueva M.O., Taylor R.C. (2020). Tyramine acts downstream of neuronal XBP-1s to coordinate inter-tissue UPR(ER) activation and behavior in C. elegans. Dev. Cell.

[7] Pellegrino M.W., Nargund A.M., Kirienko N.V., Gillis R., Fiorese C.J., Haynes C.M. (2014). Mitochondrial UPR-regulated innate immunity provides resistance to pathogen infection. Nature.

[8] Douglas P.M., Baird N.A., Simic M.S., Uhlein S., McCormick M.A., Wolff S.C., Kennedy B.K., Dillin A. (2015). Heterotypic signals from neural HSF-1 separate thermotolerance from longevity. Cell Rep..

[9] Volovik Y., Moll L., Marques F.C., Maman M., Bejerano-Sagie M., Cohen E. (2014). Differential regulation of the heat shock factor 1 and DAF-16 by neuronal nhl-1 in the nematode C. elegans. Cell Rep..

[10] Taylor R.C., Berendzen K.M., Dillin A. (2014). Systemic stress signalling: understanding the cell non-autonomous control of proteostasis. Nat. Rev. Mol. Cell Biol..

[11] Richter CP (1943). Total self-regulatory functions in animals and human beings. Harvey Lect..

[12] Li J., Labbadia J., Morimoto R.I. (2017). Rethinking HSF1 in stress, development, and organismal health. Trends Cell Biol..

[13] Morimoto R.I. (2011). The heat shock response: systems biology of proteotoxic stress in aging and disease. Cold Spring Harbor Symp. Quant. Biol..

[14] Dokladny K., Myers O.B., Moseley P.L. (2015). Heat shock response and autophagy--cooperation and control. Autophagy.

[15] Prahlad V., Cornelius T., Morimoto R.I. (2008). Regulation of the cellular heat shock response in Caenorhabditis elegans by thermosensory neurons. Science.

[16] Prahlad V., Morimoto R.I. (2011). Neuronal circuitry regulates the response of Caenorhabditis elegans to misfolded proteins. Proc. Natl. Acad. Sci. U. S. A.

[17] Maman M., Carvalhal Marques F., Volovik Y., Dubnikov T., Bejerano-Sagie M., Cohen E. (2013). A neuronal GPCR is critical for the induction of the heat shock response in the nematode C. elegans. J. Neurosci..

[18] Tatum M.C., Ooi F.K., Chikka M.R., Chauve L., Martinez-Velazquez L.A., Steinbusch H.W., Morimoto R.I., Prahlad V. (2015). Neuronal serotonin release triggers the heat shock response in C. elegans in the absence of temperature increase. Curr. Biol..

[19] Ooi F.K., Prahlad V. (2017). Olfactory experience primes the heat shock transcription factor HSF-1 to enhance the expression of molecular chaperones in C. elegans. Sci. Signal..

[20] Sugi T., Nishida Y., Mori I. (2011). Regulation of behavioral plasticity by systemic temperature signaling in Caenorhabditis elegans. Nat. Neurosci..

[21] Fawcett T.W., Sylvester S.L., Sarge K.D., Morimoto R.I., Holbrook N.J. (1994). Effects of neurohormonal stress and aging on the activation of mammalian heat shock factor 1. J. Biol. Chem..

[22] Walter P., Ron D. (2011). The unfolded protein response: from stress pathway to homeostatic regulation. Science.

[23] Ron D., Walter P. (2007). Signal integration in the endoplasmic reticulum unfolded protein response. Nat. Rev. Mol. Cell Biol..

[24] Lin J.H., Li H., Yasumura D., Cohen H.R., Zhang C., Panning B., Shokat K.M., Lavail M.M., Walter P. (2007). IRE1 signaling affects cell fate during the unfolded protein response. Science.

[25] Sun J., Liu Y., Aballay A. (2012). Organismal regulation of XBP-1-mediated unfolded protein response during development and immune activation. EMBO Rep..

[26] Sun J., Singh V., Kajino-Sakamoto R., Aballay A. (2011). Neuronal GPCR controls innate immunity by regulating noncanonical unfolded protein response genes. Science.

[27] Frakes A.E., Metcalf M.G., Tronnes S.U., Bar-Ziv R., Durieux J., Gildea H.K., Kandahari N., Monshietehadi S., Dillin A. (2020). Four glial cells regulate ER stress resistance and longevity via neuropeptide signaling in C. elegans. Science.

[28] Taylor R.C., Dillin A. (2013). XBP-1 is a cell-nonautonomous regulator of stress resistance and longevity. Cell.

[29] Daniele J.R., Higuchi-Sanabria R., Durieux J., Monshietehadi S., Ramachandran V., Tronnes S.U., Kelet N., Sanchez M., Metcalf M.G., Garcia G. (2020). UPR(ER) promotes lipophagy independent of chaperones to extend life span. Sci. Adv..

[30] Imanikia S., Sheng M., Castro C., Griffin J.L., Taylor R.C. (2019). XBP-1 remodels lipid metabolism to extend longevity. Cell Rep..

[31] Diaz-Hung M.L., Martinez G., Hetz C. (2020). Emerging roles of the unfolded protein response (UPR) in the nervous system: a link with adaptive behavior to environmental stress?. Int. Rev. Cell Mol. Biol..

[32] Shim J., Umemura T., Nothstein E., Rongo C. (2004). The unfolded protein response regulates glutamate receptor export from the endoplasmic reticulum. Mol. Biol. Cell.

[33] Costa-Mattioli M., Gobert D., Stern E., Gamache K., Colina R., Cuello C., Sossin W., Kaufman R., Pelletier J., Rosenblum K. (2007). eIF2alpha phosphorylation bidirectionally regulates the switch from short- to long-term synaptic plasticity and memory. Cell.

[34] Ounallah-Saad H., Sharma V., Edry E., Rosenblum K. (2014). Genetic or pharmacological reduction of PERK enhances cortical-dependent taste learning. J. Neurosci..

[35] Sharma V., Ounallah-Saad H., Chakraborty D., Hleihil M., Sood R., Barrera I., Edry E., Kolatt Chandran S., Ben Tabou de Leon S., Kaphzan H. (2018). Local inhibition of PERK enhances memory and reverses age-related deterioration of cognitive and neuronal properties. J. Neurosci..

[36] Trinh M.A., Kaphzan H., Wek R.C., Pierre P., Cavener D.R., Klann E. (2012). Brain-specific disruption of the eIF2alpha kinase PERK decreases ATF4 expression and impairs behavioral flexibility. Cell Rep..

[37] Liu J., Pasini S., Shelanski M.L., Greene L.A. (2014). Activating transcription factor 4 (ATF4) modulates post-synaptic development and dendritic spine morphology. Front. Cell. Neurosci..

[38] Martinez G., Vidal R.L., Mardones P., Serrano F.G., Ardiles A.O., Wirth C., Valdes P., Thielen P., Schneider B.L., Kerr B. (2016). Regulation of memory formation by the transcription factor XBP1. Cell Rep..

[39] Hayashi A., Kasahara T., Iwamoto K., Ishiwata M., Kametani M., Kakiuchi C., Furuichi T., Kato T. (2007). The role of brain-derived neurotrophic factor (BDNF)-induced XBP1 splicing during brain development. J. Biol. Chem..

[40] Altpere A., Raud S., Sutt S., Reimets R., Visnapuu T., Toots M., Vasar E. (2018). Mild stress induces brain region-specific alterations of selective ER stress markers' mRNA expression in Wfs1-deficient mice. Behav. Brain Res..

[41] Toda H., Suzuki G., Nibuya M., Shioda K., Nishijima K., Wakizono T., Kanda Y., Watanabe Y., Shimizu K., Nomura S. (2006). Behavioral stress and activated serotonergic neurotransmission induce XBP-1 splicing in the rat brain. Brain Res..

[42] Ishisaka M., Kakefuda K., Yamauchi M., Tsuruma K., Shimazawa M., Tsuruta A., Hara H. (2011). Luteolin shows an antidepressant-like effect via suppressing endoplasmic reticulum stress. Biol. Pharm. Bull..

[43] Bown C., Wang J.F., MacQueen G., Young L.T. (2000). Increased temporal cortex ER stress proteins in depressed subjects who died by suicide. Neuropsychopharmacology.

[44] Cheng D., Zhang K., Zhen G., Xue Z. (2014). The -116C/G polymorphism in XBP1 gene is associated with psychiatric illness in Asian population: a meta-analysis. Am. J. Med. Genet B Neuropsychiatr. Genet.

[45] Timberlake Ii M., Roy B., Dwivedi Y. (2019). A novel animal model for studying depression featuring the induction of the unfolded protein response in Hippocampus. Mol. Neurobiol..

[46] Kabir Z.D., Che A., Fischer D.K., Rice R.C., Rizzo B.K., Byrne M., Glass M.J., Garcia N.V.D., Rajadhyaksha A.M. (2017). Rescue of impaired sociability and anxiety-like behavior in adult cacna1c-deficient mice by pharmacologically targeting eIF2 alpha. Mol. Psychiatr..

[47] Williams K.W., Liu T., Kong X., Fukuda M., Deng Y., Berglund E.D., Deng Z., Gao Y., Liu T., Sohn J.W. (2014). Xbp1s in Pomc neurons connects ER stress with energy balance and glucose homeostasis. Cell Metabol..

[48] Cretenet G., Le Clech M., Gachon F. (2010). Circadian clock-coordinated 12 Hr period rhythmic activation of the IRE1alpha pathway controls lipid metabolism in mouse liver. Cell Metabol..

[49] Hatori M., Hirota T., Iitsuka M., Kurabayashi N., Haraguchi S., Kokame K., Sato R., Nakai A., Miyata T., Tsutsui K. (2011). Light-dependent and circadian clock-regulated activation of sterol regulatory element-binding protein, X-box-binding protein 1, and heat shock factor pathways. Proc. Natl. Acad. Sci. U. S. A.

[50] Milev N.B., Gatfield D. (2018). Circadian clocks and UPR: new twists as the story unfolds. Dev. Cell.

[51] Bu Y., Yoshida A., Chitnis N., Altman B.J., Tameire F., Oran A., Gennaro V., Armeson K.E., McMahon S.B., Wertheim G.B. (2018). A PERK-miR-211 axis suppresses circadian regulators and protein synthesis to promote cancer cell survival. Nat. Cell Biol..

[52] Gao L., Chen H., Li C., Xiao Y., Yang D., Zhang M., Zhou D., Liu W., Wang A., Jin Y. (2019). ER stress activation impairs the expression of circadian clock and clock-controlled genes in NIH3T3 cells via an ATF4-dependent mechanism. Cell. Signal..

[53] Zhu B., Zhang Q., Pan Y., Mace E.M., York B., Antoulas A.C., Dacso C.C., O'Malley B.W. (2017). A cell-autonomous mammalian 12 hr clock coordinates metabolic and stress rhythms. Cell Metabol..

[54] Majmundar A.J., Wong W.J., Simon M.C. (2010). Hypoxia-inducible factors and the response to hypoxic stress. Mol. Cell.

[55] Epstein A.C., Gleadle J.M., McNeill L.A., Hewitson K.S., O'Rourke J., Mole D.R., Mukherji M., Metzen E., Wilson M.I., Dhanda A. (2001). C. elegans EGL-9 and mammalian homologs define a family of dioxygenases that regulate HIF by prolyl hydroxylation. Cell.

[56] Bellier A., Chen C.S., Kao C.Y., Cinar H.N., Aroian R.V. (2009). Hypoxia and the hypoxic response pathway protect against pore-forming toxins in C. elegans. PLoS Pathog..

[57] Leiser S.F., Miller H., Rossner R., Fletcher M., Leonard A., Primitivo M., Rintala N., Ramos F.J., Miller D.L., Kaeberlein M. (2015). Cell nonautonomous activation of flavin-containing monooxygenase promotes longevity and health span. Science.

[58] Mehta R., Steinkraus K.A., Sutphin G.L., Ramos F.J., Shamieh L.S., Huh A., Davis C., Chandler-Brown D., Kaeberlein M. (2009). Proteasomal regulation of the hypoxic response modulates aging in C. elegans. Science.

[59] Chang A.J., Chronis N., Karow D.S., Marletta M.A., Bargmann C.I. (2006). A distributed chemosensory circuit for oxygen preference in C. elegans. PLoS Biol..

[60] Rogers C., Persson A., Cheung B., de Bono M. (2006). Behavioral motifs and neural pathways coordinating O2 responses and aggregation in C. elegans. Curr. Biol..

[61] Chang A.J., Bargmann C.I. (2008). Hypoxia and the HIF-1 transcriptional pathway reorganize a neuronal circuit for oxygen-dependent behavior in Caenorhabditis elegans. Proc. Natl. Acad. Sci. U. S. A.

[62] Kline D.D., Peng Y.J., Manalo D.J., Semenza G.L., Prabhakar N.R. (2002). Defective carotid body function and impaired ventilatory responses to chronic hypoxia in mice partially deficient for hypoxia-inducible factor 1 alpha. Proc. Natl. Acad. Sci. U. S. A.

[63] Sykiotis G.P., Bohmann D. (2010). Stress-activated cap'n'collar transcription factors in aging and human disease. Sci. Signal..

[64] Staab T.A., Griffen T.C., Corcoran C., Evgrafov O., Knowles J.A., Sieburth D. (2013). The conserved SKN-1/Nrf2 stress response pathway regulates synaptic function in Caenorhabditis elegans. PLoS Genet..

[65] Lehrbach N.J., Ruvkun G. (2016). Proteasome Dysfunction Triggers Activation of SKN-1A/Nrf1 by the Aspartic Protease DDI-1.

[66] Radhakrishnan S.K., Lee C.S., Young P., Beskow A., Chan J.Y., Deshaies R.J. (2010). Transcription factor Nrf1 mediates the proteasome recovery pathway after proteasome inhibition in mammalian cells. Mol. Cell.

[67] Blackwell T.K., Steinbaugh M.J., Hourihan J.M., Ewald C.Y., Isik M. (2015). SKN-1/Nrf, stress responses, and aging in Caenorhabditis elegans. Free Radic. Biol. Med..

[68] An J.H., Blackwell T.K. (2003). SKN-1 links C. elegans mesendodermal specification to a conserved oxidative stress response. Genes Dev..

[69] Bishop N.A., Guarente L. (2007). Two neurons mediate diet-restriction-induced longevity in C. elegans. Nature.

[70] Glover-Cutter K.M., Lin S., Blackwell T.K. (2013). Integration of the unfolded protein and oxidative stress responses through SKN-1/Nrf. PLoS Genet..

[71] Lehrbach N.J., Ruvkun G. (2019). Endoplasmic reticulum-associated SKN-1A/Nrf1 mediates a cytoplasmic unfolded protein response and promotes longevity. Elife.

[72] Paek J., Lo J.Y., Narasimhan S.D., Nguyen T.N., Glover-Cutter K., Robida-Stubbs S., Suzuki T., Yamamoto M., Blackwell T.K., Curran S.P. (2012). Mitochondrial SKN-1/Nrf mediates a conserved starvation response. Cell Metabol..

[73] Tullet J.M., Hertweck M., An J.H., Baker J., Hwang J.Y., Liu S., Oliveira R.P., Baumeister R., Blackwell T.K. (2008). Direct inhibition of the longevity-promoting factor SKN-1 by insulin-like signaling in C. elegans. Cell.

[74] Tataridas-Pallas N., Thompson M.A., Howard A., Brown I., Ezcurra M., Wu Z., Silva I.G., Saunter C.D., Kuerten T., Weinkove D. (2021). Neuronal SKN-1B modulates nutritional signalling pathways and mitochondrial networks to control satiety. PLoS Genet..

[75] Wilson M.A., Iser W.B., Son T.G., Logie A., Cabral-Costa J.V., Mattson M.P., Camandola S. (2017). skn-1 is required for interneuron sensory integration and foraging behavior in Caenorhabditis elegans. PloS One.

[76] Gallagher T., Kim J., Oldenbroek M., Kerr R., You Y.J. (2013). ASI regulates satiety quiescence in C. elegans. J. Neurosci..

[77] Khalifeh S., Oryan S., Digaleh H., Shaerzadeh F., Khodagholi F., Maghsoudi N., Zarrindast M.R. (2015). Involvement of Nrf2 in development of anxiety-like behavior by linking Bcl2 to oxidative phosphorylation: estimation in rat hippocampus, amygdala, and prefrontal cortex. J. Mol. Neurosci..

[78] Nakayama T., Okimura K., Shen J., Guh Y.J., Tamai T.K., Shimada A., Minou S., Okushi Y., Shimmura T., Furukawa Y. (2020). Seasonal changes in NRF2 antioxidant pathway regulates winter depression-like behavior. Proc. Natl. Acad. Sci. U. S. A.

[79] Zhang J.C., Yao W., Dong C., Han M., Shirayama Y., Hashimoto K. (2018). Keap1-Nrf2 signaling pathway confers resilience versus susceptibility to inescapable electric stress. Eur. Arch. Psychiatr. Clin. Neurosci..

[80] Muramatsu H., Katsuoka F., Toide K., Shimizu Y., Furusako S., Yamamoto M. (2013). Nrf2 deficiency leads to behavioral, neurochemical and transcriptional changes in mice. Gene Cell..

